# Calpain Inhibition Restores Autophagy and Prevents Mitochondrial Fragmentation in a Human iPSC Model of Diabetic Endotheliopathy

**DOI:** 10.1016/j.stemcr.2019.01.017

**Published:** 2019-02-21

**Authors:** Sang-Bing Ong, Won Hee Lee, Ning-Yi Shao, Nur Izzah Ismail, Khairunnisa Katwadi, Mim-Mim Lim, Xiu-Yi Kwek, Nathaly Anto Michel, Jiajun Li, Jordan Newson, Soroush Tahmasebi, Jalees Rehman, Kazuki Kodo, Hye Ryoun Jang, Sang-Ging Ong

**Affiliations:** 1 Signature Research Program in Cardiovascular & Metabolic Disorders, Duke-NUS Medical School, Singapore 169857, Singapore; 2 Department of Basic Medical Sciences, University of Arizona, Phoenix, AZ 85004, USA; 3 Stanford Cardiovascular Institute, Stanford University School of Medicine, Stanford, CA 94305, USA; 4 Department of Biomedical Engineering, Faculty of Engineering, University of Malaya, 50603 Kuala Lumpur, Malaysia; 5 Department of Pharmacology, The University of Illinois College of Medicine, Chicago, IL 60612, USA; 6 Division of Cardiology, Department of Medicine, The University of Illinois College of Medicine, 909 S Wolcott Avenue, Chicago, IL 60612, USA; 7 Department of Pediatrics, Keio University School of Medicine, Tokyo 160-8582, Japan; 8 Division of Nephrology, Department of Medicine, Samsung Medical Center, Sungkyunkwan University School of Medicine, 81 Irwon-ro, Gangnam-gu, Seoul 06351 Republic of Korea

**Keywords:** diabetes, endothelial dysfunction, iPSC, iPSC-ECs, calpain, autophagy, mitochondrial morphology, ischemia-reperfusion injury

## Abstract

The relationship between diabetes and endothelial dysfunction remains unclear, particularly the association with pathological activation of calpain, an intracellular cysteine protease. Here, we used human induced pluripotent stem cells-derived endothelial cells (iPSC-ECs) to investigate the effects of diabetes on vascular health. Our results indicate that iPSC-ECs exposed to hyperglycemia had impaired autophagy, increased mitochondria fragmentation, and was associated with increased calpain activity. In addition, hyperglycemic iPSC-ECs had increased susceptibility to cell death when subjected to a secondary insult—simulated ischemia-reperfusion injury (sIRI). Importantly, calpain inhibition restored autophagy and reduced mitochondrial fragmentation, concurrent with maintenance of ATP production, normalized reactive oxygen species levels and reduced susceptibility to sIRI. Using a human iPSC model of diabetic endotheliopathy, we demonstrated that restoration of autophagy and prevention of mitochondrial fragmentation via calpain inhibition improves vascular integrity. Our human iPSC-EC model thus represents a valuable platform to explore biological mechanisms and new treatments for diabetes-induced endothelial dysfunction.

## Highlights

• iPSC-ECs as a novel model of human diabetic endotheliopathy
• Hyperglycemic iPSC-ECs have impaired autophagy and increased mitochondria scission
• Calpain inhibition reverses detrimental effects of hyperglycemia on iPSC-ECs
• iPSC-ECs can be used to study comorbidities—hyperglycemia with sIRI

## Teaser

In this article, Ong and colleagues used human induced pluripotent stem cell-derived endothelial cells (iPSC-ECs) as a novel disease modeling platform to study the effects of diabetes on vascular dysfunction and provided the cause for calpain inhibitors as an anti-diabetic and cardioprotective drug.

## Introduction

Diabetes mellitus is a complex metabolic disease affecting more than 300 million people worldwide (American Diabetes Association, 2014, Danaei et al., 2011). The most common form is type 2 diabetes, characterized by a lack of response to insulin in the peripheral tissues as well as impaired secretion of insulin (Ako et al., 2006, American Diabetes Association, 2014). The progression of diabetes mellitus is closely associated with increased risk of cardiovascular disorders such as ischemia-reperfusion injury (IRI), generally attributed to the adverse effects of hyperglycemia and oxidative stress (Gerich, 2007, Kalofoutis et al., 2007, Martin-Timon et al., 2014).

Endothelial dysfunction constitutes an early hallmark associated with cardiovascular disorders in diabetes. Endothelial cells (ECs) have significantly impaired ability to promote vasodilation, fibrinolysis, and anti-aggregation to the point of developing atherosclerosis (Hadi and Suwaidi, 2007, Sena et al., 2013). Calpains, a 15-member family of Ca^2+^-dependent non-lysosomal cysteine proteases, which include the ubiquitous calpain-1 and -2 (*μ*- and *m*-calpains) have been reported to be pathologically activated in diabetes mellitus, although the links to hyperglycemia-induced, mitochondrial-mediated endothelial dysfunction is not fully understood. Calpain over-activation has been shown to lead to EC dysfunction and inflammatory responses (Goll et al., 2003, Potz et al., 2016, Stalker et al., 2005, Suzuki et al., 2004, Vindis et al., 2005)—for instance, calpain-1 activation promotes hyperhomocysteinemia-induced extracellular matrix remodeling mediated by matrix metalloproteinase 9, leading to elevated levels of reactive oxygen species (ROS) and vascular damage (Moshal et al., 2006, Papatheodorou and Weiss, 2007). Likewise, inhibition of calpain has been reported to be linked to reduced oxidative stress and attenuation of endothelial dysfunction in diabetes (Chen et al., 2014). On this background, perturbations in mitochondrial function have been demonstrated to govern cell fate (Galluzzi et al., 2012, Granville and Gottlieb, 2002, McBride et al., 2006, Ong and Gustafsson, 2012, Parone et al., 2002). In the setting of hyperglycemia, the dysregulation of both autophagy and mitochondrial morphology have been associated with impairment of normal cellular function and intolerance to injury (Fetterman et al., 2016, Jheng et al., 2012, Jung et al., 2008, Lv et al., 2014, Yu et al., 2008). However, the association between calpain activation and mitochondrial aberration in the pathophysiology of hyperglycemic-induced endothelial dysfunction remains to be fully elucidated.

Ongoing studies have generated and utilized different cellular and animal models of diabetes to study the underlying mechanisms of hyperglycemic disorders. However, utilization of patient-specific biological material could significantly enhance our knowledge of the precise mechanisms behind diabetes complications. Although a small number of human EC lines and primary human cells have contributed significantly to the understanding of vascular biology in the context of diabetes, the difficulty in obtaining vascular tissues, especially in patients with specific genetic backgrounds remains a limitation. Recent efforts have focused on generating patient-specific material using induced pluripotent stem cells (iPSCs) that can be derived from human somatic cells, then differentiated into desired lineages including iPSC-derived endothelial cells (iPSC-ECs) (Sone et al., 2007, Takahashi et al., 2007, Takahashi and Yamanaka, 2006, Taura et al., 2009) or cardiomyocytes (iPSC-CMs) for disease modeling (Burridge et al., 2016, Kodo et al., 2016). Human iPSC-ECs represent a wide spectrum of usage including cell-based therapy, disease modeling, and drug screening. For example, patient-specific iPSC-ECs have been used to successfully recapitulate *in vitro* the clinical phenotype of pulmonary arterial hypertension and fibrodysplasia ossificans progressiva (Barruet et al., 2016, Gu et al., 2017).

Here, we utilized human iPSC-ECs as a model of diabetic endotheliopathy and sought to understand hyperglycemia-induced changes to autophagy and mitochondrial dynamics as potential pathophysiological factors leading to endothelial dysfunction. We demonstrated that exposure of iPSC-ECs to hyperglycemia led to impaired vascular health as indicated by disrupted tube formation, increased oxidative stress, and decreased ATP. We also showed that hyperglycemia resulted in decreased autophagy as well as increased mitochondrial fragmentation in iPSC-ECs. The aforementioned effects may be attributed, in part, to increased calpain activation, as inhibition of calpain using MDL-28170 resulted in successful reversal. Lastly, hyperglycemic iPSC-ECs were more susceptible to cell death when exposed to simulated ischemia-reperfusion injury (sIRI) demonstrating the use of iPSC-ECs for investigating comorbidities.

## Results

### *In Vitro* Monolayer Endothelial Differentiation of Human iPSC

**Figure 1:**
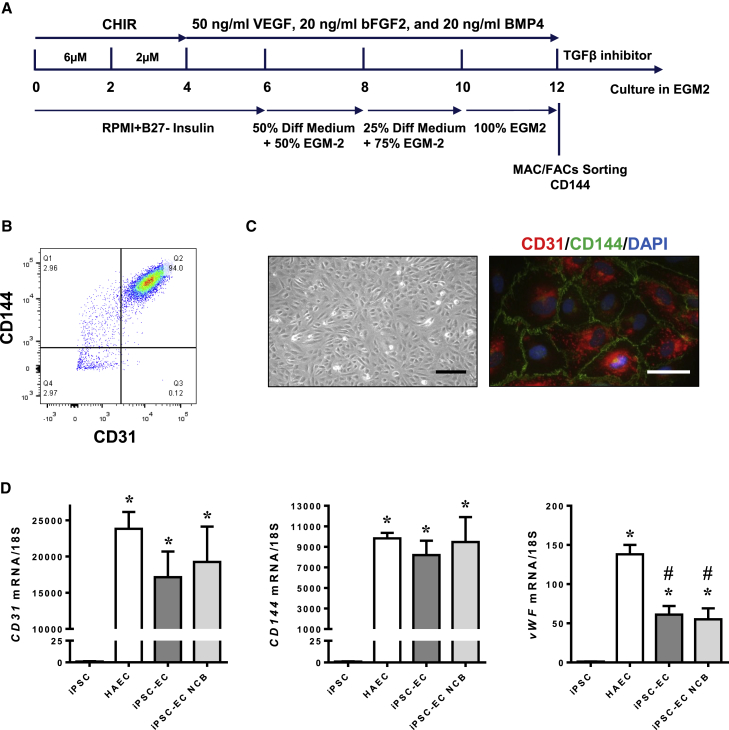
Characterization of iPSC-ECs (A) A brief scheme of iPSC endothelial differentiation. (B) Representative flow cytometry analysis of purified iPSC-ECs stained for both CD144 and CD31. ECs that were isolated using CD144 MicroBeads were also positive for CD31, a commonly used EC marker. (C) iPSC-ECs demonstrated a cobblestone-like morphology and stained positive for both CD31 (red) and CD144 (green). Scale bars, 200 μm (left panel) and 50 μm (right panel). (D) qPCR of endothelial-markers compared with iPSCs generated using the current protocol (iPSC-EC), from an alternative protocol (iPSC-EC NCB) and HAECs. Data are represented as means ± SEM (N = 4 independent experiments performed in triplicates, ^∗^p < 0.05 versus iPSC, ^#^p < 0.05 versus HAEC).

Human iPSCs were differentiated into ECs as described previously (Gu et al., 2017). In brief, cells were treated with CHIR (6 μM) from day 0 to 2, followed by CHIR (2 μM) from day 2 to 4 for mesoderm induction. From day 4 to 12 of differentiation, cells were treated with vascular endothelial growth factor (VEGF), basic fibroblast growth factor (FGF), and bone morphogenetic protein 4 (BMP4) in EGM-2 endothelial growth medium to promote specification to ECs. On day 12 of differentiation, iPSC-ECs were purified by magnetic-activated cell sorting for EC surface marker CD144 (Figure 1A). Fluorescence-activated cell sorting analysis showed that ∼90% of the sorted CD144-positive cells were also positive for another endothelial marker, CD31 (Figure 1B), and the purity of iPSC-ECs generated using this protocol was comparable with iPSC-ECs generated using an alternative protocol (Patsch et al., 2015) and against primary human aortic endothelial cells (HAECs) (Figure S1A). These cells exhibited a typical cobblestone morphology of ECs and stained positive for both CD31 and CD144 (Figures 1C and S1B). Compared with undifferentiated iPSCs, iPSC-ECs from both differentiation protocols exhibited robust expression of endothelial marker genes *CD31*, *CD144*, and *von Willebrand Factor* (*vWF*), although the expression of *vWF* in these iPSC-ECs was lower than HAECs, confirming successful differentiation of iPSC-ECs (Figure 1D). Similar to HAECs, differentiated iPSC-ECs also took up acetylated low-density lipoprotein (Figure S1C) and expressed *vascular cell adhesion molecule 1* (*VCAM1*) when treated with tumor necrosis factor alpha, a proinflammatory cytokine (Figure S1D), further verifying these cells as bona fide iPSC-ECs.

### Exposure of iPSC-ECs to Hyperglycemia Induces Endothelial Dysfunction

**Figure 2:**
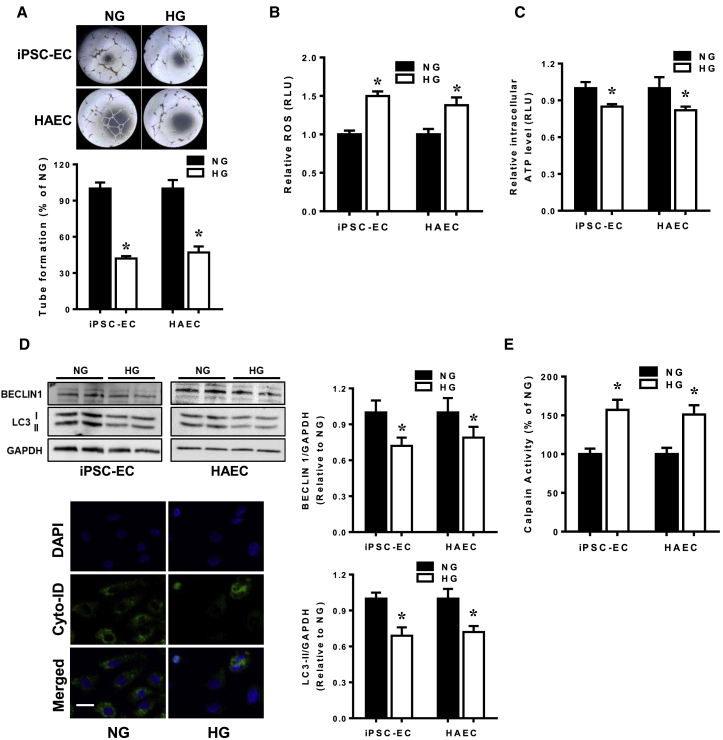
Exposure of iPSC-ECs to Hyperglycemia Leads to Endothelial Dysfunction (A) Representative bright-field images of tube formation (angiogenic capability) of iPSC-ECs subjected to normoglycemic (NG) or hyperglycemic (HG) treatments. Quantitative data from the tube formation assay (lower panel) revealed reduced tube formation of both iPSC-ECs and HAECs in HG expressed as a percentage against cells in NG (control). Mean ± SEM of four independent experiments, ^∗^p < 0.05 versus NG cells. (B and C) iPSC-ECs and HAECs exposed to HG had higher oxidative stress (B) and reduced ATP (C) compared with NG control. Data are represented as means ± SEM of four independent experiments, ^∗^p < 0.05 versus NG cells. (D) Representative immunoblot (top left panel) and densitometry analysis (right panel) of autophagy-related proteins BECLIN 1 and LC3-II normalized against GAPDH in iPSC-ECs and HAECs subjected to NG or HG treatment. Data are represented as means ± SEM of four independent experiments, ^∗^p < 0.05 versus NG cells. Cyto-ID staining of autophagosome formation in iPSC-ECs subjected to NG or HG treatment is also shown. Scale bar, 50 μm. (E) Measurement of calpain activity in iPSC-ECs and HAECs grown in HG versus HG, normalized against the percentage in NG-treated cells. Data are represented as mean ± SEM of three independent experiments, ^∗^p < 0.05 versus NG cells.

As lack of neovascularization and vasculogenesis induced by hyperglycemia often precede the progression of diabetic macrovascular complications, we first investigated the effects of hyperglycemia on the endothelial differentiation potential of iPSCs and differentiated iPSC-ECs. The presence of hyperglycemia during endothelial differentiation impaired the generation of CD144-positive ECs, suggesting that hyperglycemia causes inhibitory effects on the conversion of stem/progenitor cells into ECs (Figure S2A). Notwithstanding the reduced endothelial differentiation potential, differentiated iPSC-ECs cultured under hyperglycemic conditions did not appear to be morphologically distinct compared with normoglycemic controls (Figure S2B), although the proliferative capacity of iPSC-ECs under hyperglycemia was significantly reduced compared with normoglycemic iPSC-ECs (Figure S2C). These subtle differences suggest that hyperglycemia predisposes ECs toward a diabetic phenotype. We next assessed the tube formation potential of iPSC-ECs and found that iPSC-ECs exposed to hyperglycemia had significantly impaired tube formation ability compared with cells in normoglycemia (Figure 2A), results which were corroborated using HAECs. Hyperglycemic conditions also resulted in increased oxidative stress (Figure 2B) and reduced ATP levels (Figure 2C) compared with normoglycemic conditions in both iPSC-ECs and HAECs. Measurement of autophagic levels by immunoblotting revealed lower levels of both BECLIN 1 and LC3-II in iPSC-ECs and HAECs exposed to hyperglycemia, further supported by reduced presence of autophagosomes compared with iPSC-ECs in normoglycemic condition (Figure 2D), demonstrating an impairment in autophagy. Importantly, hyperglycemia led to a significant increase in calpain activity in both iPSC-ECs and HAECs (Figure 2E), which has previously been shown to be associated with vascular dysfunction (Miyazaki et al., 2011).

### Calpain Inhibition Rescues Endothelial Dysfunction in Hyperglycemic iPSC-ECs

**Figure 3:**
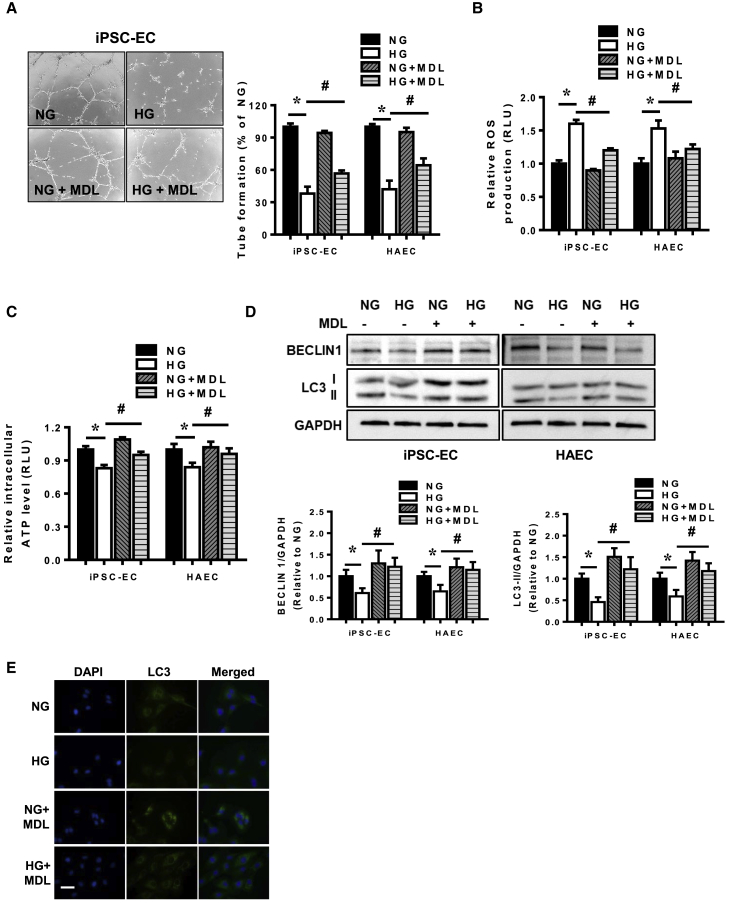
Calpain Inhibition Restores Endothelial Dysfunction in Hyperglycemic iPSC-ECs (A) Tube formation assays were performed to assess angiogenic capacity of iPSC-ECs and HAECs treated with vehicle or MDL-28170 (MDL) and exposed to normoglycemia (NG) or hyperglycemia (HG). Representative bright-field images of tube formation extent in iPSC-ECs in NG or HG, with or without MDL-28170 (left panel). Data are expressed as a percentage of tube formation in NG iPSC-ECs or HAECs and represent a means ± SEM of four independent experiments, ^∗^p < 0.05 versus NG cells, ^#^p < 0.05 versus HG cells. (B and C) Measurement of ROS levels (B) and ATP levels (C) in iPSC-ECs and HAECs treated with vehicle or MDL-28170 and exposed to NG or HG. Data are represented as means ± SEM of four independent experiments, ^∗^p < 0.05 versus NG cells, ^#^p < 0.05 versus HG cells. (D) Measurement of autophagy levels by immunoblotting in iPSC-ECs and HAECs treated with or without MDL-28170 under NG or HG conditions. Data are represented as means ± SEM of four independent experiments, ^∗^p < 0.05 versus NG cells, ^#^p < 0.05 versus HG cells. (E) Cyto-ID staining of autophagosomes in NG or HG iPSC-ECs treated with or without MDL-28170.

Having shown that exposure of iPSC-ECs to hyperglycemia led to endothelial dysfunction and was associated with increased calpain activity, we examined whether inhibition of calpain is beneficial for restoring endothelial health. Treatment of iPSC-ECs and HAECs with MDL-28170, a potent calpain I and II inhibitor (Mehdi, 1991), led to decreased calpain activity (data not shown) and improved tube formation in cells exposed to hyperglycemia (Figures 3A and S3). Likewise, whereas the presence of MDL-28170 did not modify ROS production in iPSC-ECs nor HAECs under normoglycemic conditions, MDL-28170 successfully reduced hyperglycemia-induced ROS production significantly (Figure 3B). This is in line with the ATP production whereby impairment of ATP production that occurred in the presence of hyperglycemia was significantly reversed by calpain inhibition (Figure 3C). Inhibition of calpain also restored the levels of autophagy in both iPSC-ECs and HAECs exposed to hyperglycemia marked by higher levels of BECLIN 1 and LC3-II normalized against GAPDH (Figure 3D) and increased formation of autophagosomes (Figure 3E).

### Hyperglycemia-Induced Mitochondrial Fragmentation in iPSC-ECs Is Reversed by Calpain Inhibition

**Figure 4:**
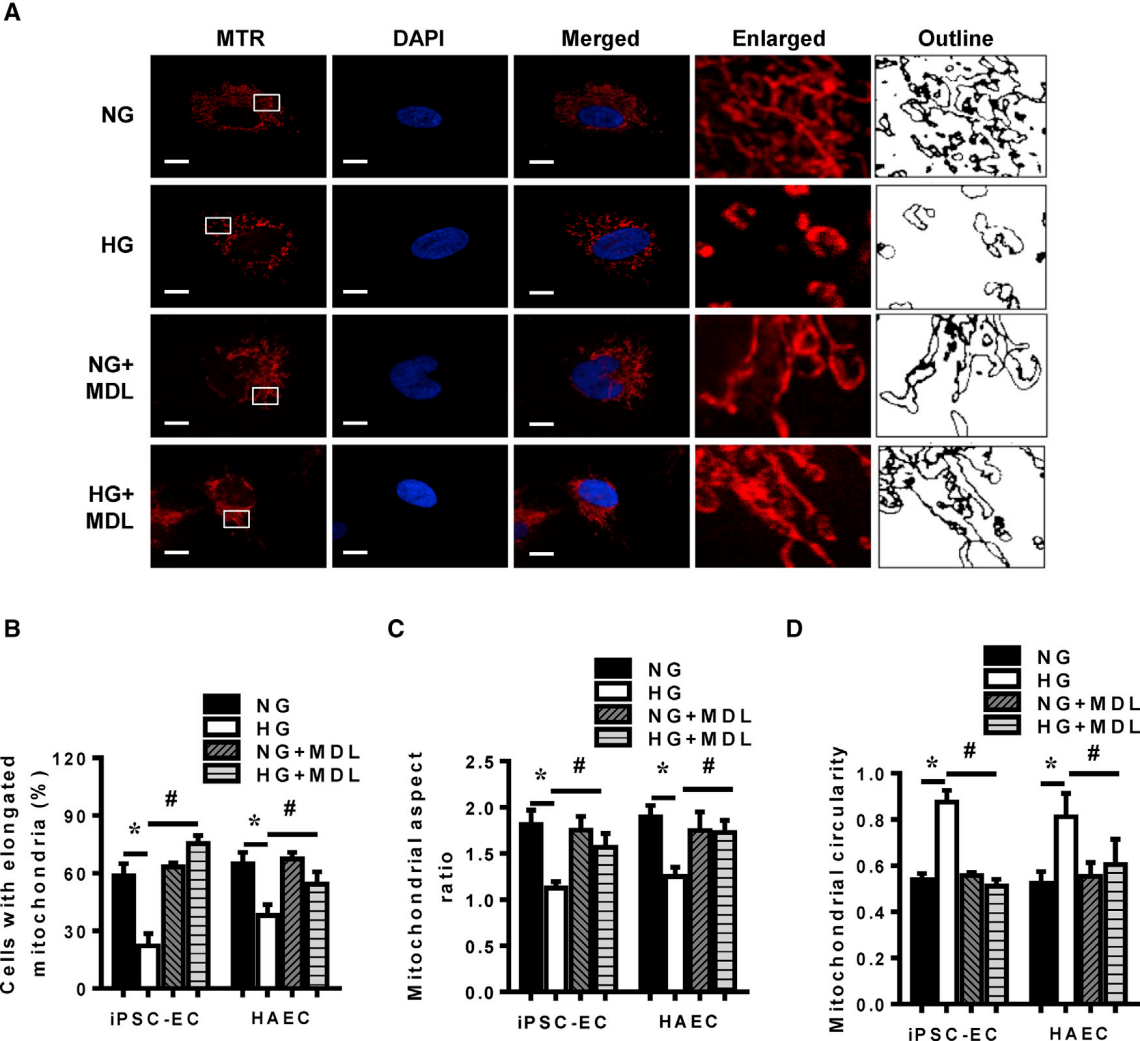
Mitochondrial Morphology of iPSC-ECs Following Hyperglycemic Treatment in the Presence of MDL-28170 (A) Representative confocal images and Fiji/ImageJ outline tracings of iPSC-ECs in either normoglycemia (NG) or hyperglycemia (HG) environments, with or without MDL-28170 (MDL), stained with MitoTracker Red (MTR) and DAPI for the nucleus. Scale bars, 10 μm. (B) Proportion of iPSC-ECs and HAECs with elongated mitochondria in either NG or HG environment, with or without MDL-28170, data are represented as means ± SEM of four independent experiments, ^∗^p < 0.05 versus NG cells, ^#^p < 0.05 versus HG cells. (C) Assessment of mitochondrial aspect ratio in iPSC-ECs and HAECs cultured in an either NG or HG environment, with or without MDL-28170, using Fiji/ImageJ. Data are represented as means ± SEM of four independent experiments, ^∗^p < 0.05 versus NG cells, ^#^p < 0.05 versus HG cells. (D) Measurement of mitochondrial circularity in iPSC-ECs and HAECs in an NG or HG environment, with or without MDL-28170, using Fiji/ImageJ. Data are represented as means ± SEM of four independent experiments, ^∗^p < 0.05 versus NG cells, ^#^p < 0.05 versus HG cells.

Prior studies have demonstrated aberrant fragmentation of the mitochondria under hyperglycemic conditions in a variety of cell types (Jheng et al., 2012, Yu et al., 2008, Yu et al., 2017). Using MitoTracker Red staining of mitochondria, we observed normoglycemic iPSC-ECs to have predominantly elongated mitochondria, and exposure of these cells to hyperglycemia led to fragmentation and dispersion of elongated mitochondria around the cytosol (Figure 4A, upper two rows). Quantitative analysis of iPSC-ECs showed that predominantly elongated mitochondria were significantly reduced in hyperglycemic conditions versus normoglycemia (Figure 4B). Particle analysis of the images using Fiji/ImageJ revealed mitochondria to have lower aspect ratio (AR) (Figure 4C) and higher circularity values (Figure 4D) under hyperglycemic conditions indicating hyperglycemic mitochondria undergoing fragmentation and becoming punctate. Importantly, HAECs subjected to hyperglycemia also had reduced number of cells with elongated mitochondria (Figure 4B), marked by lower mitochondrial AR (Figure 4C) and higher circularity values (Figure 4D) similar to iPSC-ECs. As we observed calpain activation in iPSC-ECs exposed to hyperglycemia and calpain has been shown to regulate mitochondrial dynamics previously in other cell types, we were interested in further evaluation of the association between hyperglycemia and induction of mitochondrial fission. To test whether calpain activation following hyperglycemia is responsible for induction of mitochondrial fragmentation, the mitochondrial morphology following calpain inhibition was studied. Importantly, we observed that inhibition of calpain using MDL-28170 prevented the fragmentation of mitochondria in iPSC-ECs induced by hyperglycemia with the number of iPSC-ECs with predominantly elongated mitochondria significantly increased (Figure 4A, lower two rows and B), an effect corroborated using Fiji/ImageJ-particle analysis (Figures 4C and 4D). Inhibition of calpain in HAECs also had similar effects (Figures 4B–4D). MDL-28170 in iPSC-ECs grown under normoglycemic conditions did not significantly alter the mitochondrial morphology.

### Gene Expression Profiling of iPSC-ECs Subjected to Hyperglycemia

**Figure 5:**
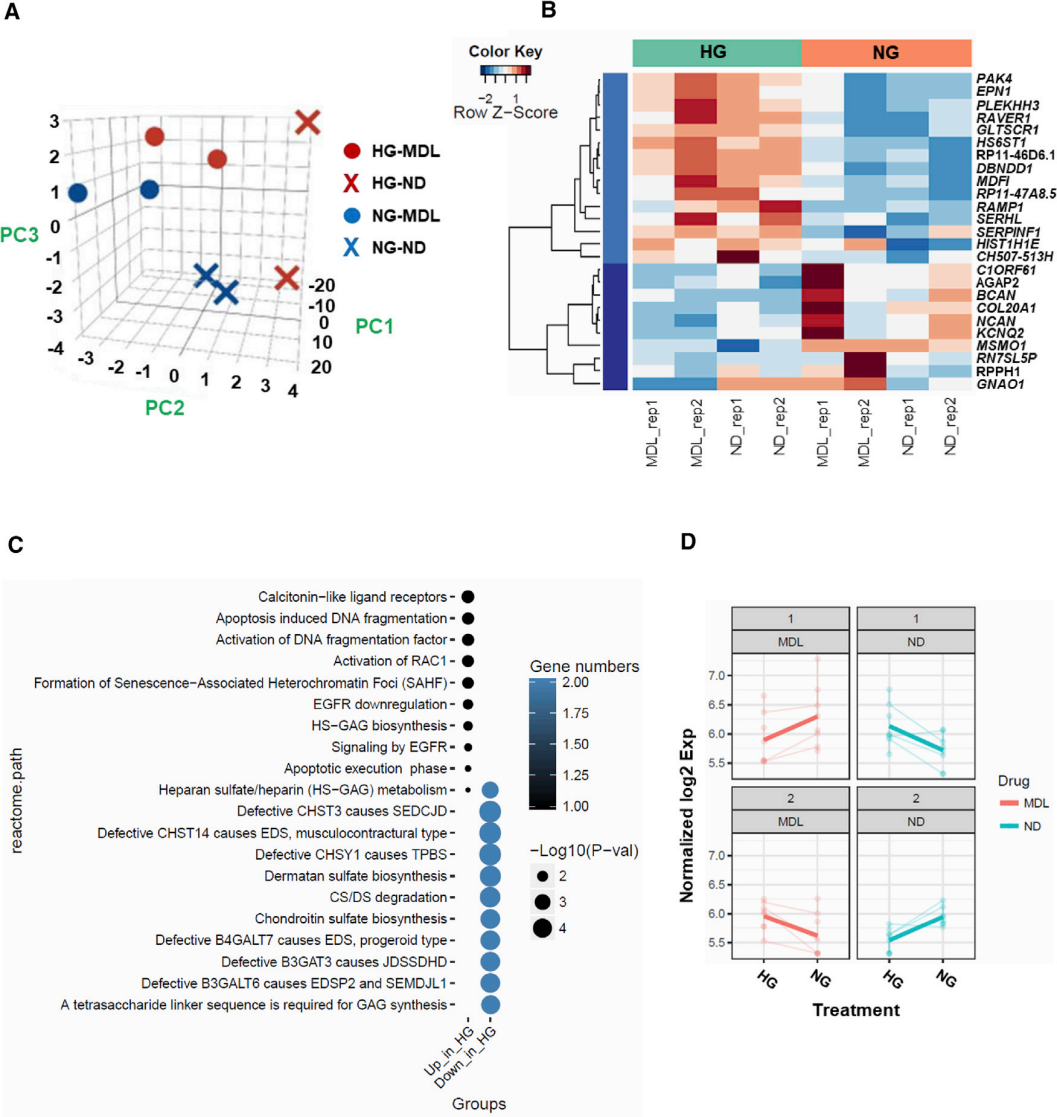
Transcriptomic Analysis of iPSC-ECs Treated with MDL-28170 (A) Principal component analysis of gene expression across two different iPSC-ECs under normo- or hyperglycemia and treated with either vehicle or MDL-28170. (B) Heatmap of differentially expressed genes in iPSC-ECs exposed to NG or HG, with or without MDL-28170. (C) Pathway analysis of differentially expressed genes in hyperglycemic iPSC-ECs. (D) Line plots of differentially expressed genes clusters between glucose and MDL treatments.

For an in-depth understanding on the effects of both hyperglycemia and calpain inhibition, we then performed RNA sequencing (GEO accession number: GSE123699) and analyzed the global expression pattern changes among these factors—normo-/hyperglycemia and non-treatment/MDL-28170-treated. All genes were normalized by the sequencing depth, log transformed, and variance stabilizing transformed. The principal component analysis of all genes indicated that the genetic background contributed the most variance (96%) in principal component 1 (PC1), while hyperglycemia and MDL treatments contributed to the variances of PC2 (2%) and PC3 (1%), respectively (Figure 5A). We identified 25 differentially expressed genes (DEGs) in the hyperglycemia condition (Figure 5B), with 15 genes upregulated in hyperglycemic group, whereas 10 genes were downregulated in the normoglycemic group. Functional enrichment analysis of the DEGs is shown in Figure 5C, which includes several pathways related to apoptosis such as apoptosis-induced DNA fragmentation, activation of DNA fragmentation factor, and apoptotic execution phase, which support our findings that hyperglycemia induces endothelial dysfunction, possibly ultimately leading to apoptosis. In addition, pathway analysis also revealed formation of senescence-associated heterochromatin foci, indicating that ECs exposed to hyperglycemia may undergo premature senescence, thus affecting cellular proliferation, representing an alternative pathway which may be explored in the future. We also attempted to identify the genes that had differential changes between both hyperglycemia and MDL treatments simultaneously, but only eight genes were detected (Figure 5D).

### Hyperglycemic iPSC-ECs Have Increased Susceptibility against sIRI that Is Reversed by Calpain Inhibition

**Figure 6:**
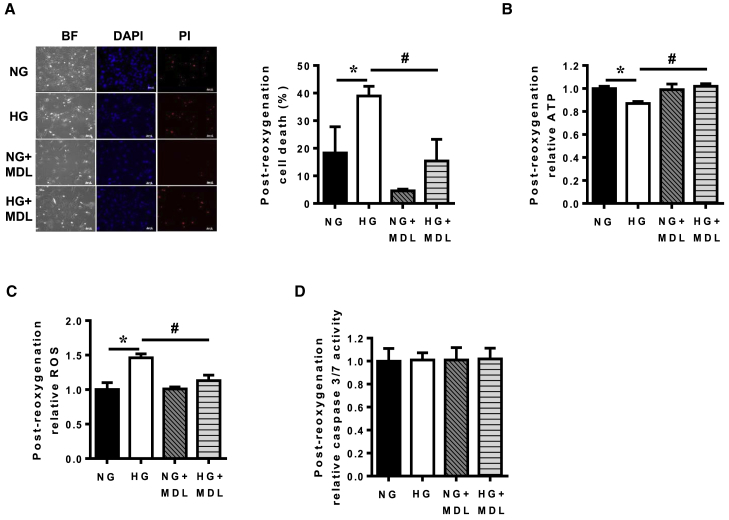
Hyperglycemic iPSC-ECs Displayed Increased Susceptibility against Simulated Ischemia Reperfusion Injury that Can Be Prevented by Calpain Inhibition (A) Post-reoxygenation cell viability of normoglycemia (NG) or hyperglycemia (HG) iPSC-ECs, with or without MDL-28170, following exposure to sIRI was determined by propidium iodide (PI) staining, as visualized by fluorescence microscopy. Data are represented as means ± SEM of four independent experiments, ^∗^p < 0.05 versus NG cells, ^#^p < 0.05 versus HG cells. Scale bars, 100 μm. (B) ATP measurement in NG- or HG-treated iPSC-ECs following post-reoxygenation, with or without MDL-28170. Data are represented as means ± SEM of four independent experiments, ^∗^p < 0.05 versus NG cells, ^#^p < 0.05 versus HG cells. (C) Measurement of ROS in NG- or HG-treated iPSC-ECs following sIRI, with and without MDL-28170. Data are represented as means ± SEM of four independent experiments, ^∗^p < 0.05 versus NG cells, ^#^p < 0.05 versus HG cells. (D) Measurement of caspase-3/7 activity in NG- or HG-treated iPSC-ECs following sIRI, with and without MDL-28170. Data are represented as means ± SEM of four independent experiments.

Diabetic patients often have increased risk of developing additional comorbidities such as ischemia and stroke. To investigate if diabetic iPSC-ECs were more susceptible to IRI, we subjected iPSC-ECs in both normoglycemic and hyperglycemic conditions to sIRI and measured cell death, ATP production, and ROS levels. Our results demonstrated that iPSC-ECs in hyperglycemia had significantly increased cell death (Figure 6A), lower ATP levels (Figure 6B), and higher oxidative stress (Figure 6C) compared with normoglycemic iPSC-ECs when subjected to sIRI. In accordance with earlier results, inhibition of calpain activity via MDL-28170 successfully reduced cell death (Figure 6A), improved ATP levels (Figure 6B), and lowered ROS levels (Figure 6C) in iPSC-ECs in hyperglycemic conditions following exposure to sIRI. To further corroborate the role of mitochondria in determining the iPSC-ECs fate in the hyperglycemic environment, we analyzed the relative levels of caspase-3/7 between the different treatments in the cells. No difference in caspase-3/7 level was detected across the groups (Figure 6D).

## Discussion

In the present study, we successfully demonstrated that human iPSC-ECs can be used as a valuable disease-modeling platform to assess endothelial dysfunction under a diabetic-like environment. We observed that hyperglycemia resulted in iPSC-ECs undergoing endothelial dysfunction, which was associated with enhanced calpain activity, impaired autophagy, and increased mitochondrial fragmentation. Blockade of calpain activity using a calpain inhibitor, MDL-28170, successfully reversed these detrimental effects. Although previous studies have demonstrated a relationship between calpain inhibition and induction of autophagy in neurodegenerative disease (Watchon et al., 2017), and that diabetes is associated with calpain activation (Chen et al., 2014) and also impaired autophagy (Fetterman et al., 2016), our study successfully established a direct link between hyperglycemia-induced autophagy impairment and perturbed mitochondrial dynamics with aberrant activation of calpain in ECs. An additional strength of our study lies in the use of human iPSC-ECs as a novel model for understanding the pathobiology associated with hyperglycemia, as these individual-specific cells retain the genetic and epigenetic backgrounds of the donors and can potentially be used in the future to study the effects of patient-specific mutations under hyperglycemia through genome editing using isogenic lines. This is further supported by our results demonstrating that iPSC-ECs can recapitulate the detrimental effects of hyperglycemia in terms of calpain activation, increased oxidative stress, and impaired autophagy seen in primary HAECs. Interestingly, the pattern of autophagy impairment in HAECs from a separate study that showed only an increase in P62 without a decrease in LC3-II (Fetterman et al., 2016), which may be due to the difference in hyperglycemic stimulus including the inclusion of endothelin-1 in our hyperglycemic conditions, a factor known to be increased in diabetic patients (Schneider et al., 2002), as well as a longer treatment duration in our study. In addition, our model also revealed that hyperglycemic iPSC-ECs had increased susceptibility compared with control iPSC-ECs when exposed to a secondary stress (sIRI), and that calpain inhibition mitigated the deleterious effects further expanding the use of iPSC-ECs for studying comorbidities.

The detrimental effects of hyperglycemia encompasses reduced autophagy (Fetterman et al., 2016, Hsu et al., 2016), mitochondrial fragmentation (Jheng et al., 2012, Yu et al., 2008, Yu et al., 2011), loss of ATP production, and increased levels of ROS (Watanabe et al., 2014, Yu et al., 2006, Yu et al., 2011), all of which can be observed in the diabetic heart, leading to an increased susceptibility to IRI. Autophagy reduction and mitochondrial fragmentation have also been previously linked to increased injury following IRI in the heart (Ma et al., 2015, Ong et al., 2010). Nonetheless, we now demonstrate this in the setting of diabetic iPSC-ECs. Calpain over-activation has been observed in the settings of streptozotocin-induced diabetes and hyperglycemic cardiomyocytes (Li et al., 2009). Over-activation of mitochondrial calpain-1 in type 1 diabetic murine heart and cultured cardiomyocytes generates superoxide while negatively regulating ATP5A1 protein, leading to ATP synthase disruption (Ni et al., 2016). High-fat diet and the resulting obesity also induces over-activation of calpain-2, causing cleavage and degradation of autophagy-related protein 7 and Beclin1, resulting in inhibition of autophagy (Kim et al., 2008, Yang et al., 2010). Calpain activation following exposure to pro-apoptotic stimuli also cleaves Atg5, prompting the translocation of the truncated Atg5 from the cytosol to the mitochondria and stimulating apoptotic cell death via the association with the antiapoptotic molecule Bcl-xL (Lepine et al., 2011, Shi et al., 2013, Yousefi et al., 2006). It is worthwhile noting that our study did not differentiate between the ubiquitously expressed calpain-1 and calpain-2 isoforms. Calpain-1, found in the mitochondrial intermembrane space acts to increase apoptosis-inducing factor cleavage in cardiac mitochondria during ischemia-reperfusion (Chen et al., 2011), while calpain-2, localized to the mitochondrial matrix, participates in mitochondrial permeability transition pore opening and inactivation of complex I following ischemia-reperfusion (Shintani-Ishida and Yoshida, 2015, Thompson et al., 2016), is activated following large calcium fluxes which occurs during apoptosis (Goll et al., 2003) and mediates β cell dysfunction and apoptosis in type 2 diabetes (Huang et al., 2010), settings which are equally relevant in our study.

The effect of calpain inhibition of mitochondrial morphology suggests that fragmented mitochondria may be associated with impairment of the autophagic machinery to remove the damaged mitochondria, whereas the presence of predominantly elongated mitochondria is suggestive of successful clearance of damaged or dysfunctional mitochondria. The inhibition of mitochondrial fission has previously been demonstrated to protect against IRI in the heart, which also aligns well with the findings of this study, implicating a role of calpain in interfering with the balance in mitochondrial dynamics in iPSC-ECs (Breckenridge et al., 2003, Estaquier and Arnoult, 2007, Guo et al., 2014, Ong et al., 2010, Ong et al., 2015, Xie et al., 2013). Whether the level of calpain is further induced in the combination of hyperglycemia and hypoxia as in our study remains to be clarified.

Another important aspect of our study lies in establishing the effects of calpain inhibition in iPSC-ECs subjected to sIRI. Calpain translocation from the cytosol to the mitochondria occurs during ischemia, whereas reperfusion allows for its activation (Hernando et al., 2010). Intuitively, the calpain inhibitor should be administered at the onset of reperfusion. Nonetheless, it is noteworthy to point out that the ischemic insult is sufficient to induce mitochondrial fragmentation (Ong et al., 2010), thus necessitating the presence of an inhibitor during the ischemic period itself. Furthermore, the hyperglycemic environment in the iPSC-ECs culture necessitates the presence of the calpain inhibitor pre-ischemia to properly elucidate the effects of calpain activation. Thus, in our study, the calpain inhibitor was present pre-ischemia, throughout ischemia, as well as during reoxygenation. Our results also suggest that the cell death in this model of diabetic endotheliopathy may be caspase independent and mitochondrial dependent, although studying the time response of the cells undergoing ischemia and reperfusion, as well as the application of modulators of necroptosis, may elicit clearer details. Of note, the iPSC-ECs used in this proof-of-concept study were derived from healthy volunteers and not from diabetic patients with a polygenic background. However, the successful recapitulation of endothelial dysfunction in these healthy iPSC-ECs when exposed to hyperglycemia will allow future work utilizing cells derived from patients with a polygenic background for more precise disease modeling. Gene expression profiling, which revealed association of senescence-related pathways in iPSC-ECs exposed to hyperglycemia, also revealed additional targets for preventing hyperglycemia-induced endothelial dysfunction; this is supported by our data that demonstrated reduced proliferation of iPSC-ECs following exposure to hyperglycemia, although more definitive experiments are needed to confirm the involvement of these pathways.

In summary, we provide evidence for the association between calpain inhibition and conservation of mitochondrial function with regard to autophagy and mitochondrial morphology, which ultimately leads to enhanced tolerance to cardiac injury in the settings of diabetes. These findings in the iPSC-ECs support a novel model for the study of diabetic angiopathy (micro- or macroangiopathy), which may have significant implications in diabetic cardiac complications. The findings also shed further light on the mechanisms underlying the benefits of calpain inhibition as a therapeutic target in tackling cardiac disorders.

## Experimental Procedures

Experimental methods are also provided in Supplemental Information.

### Cell Culture

Primary HAECs were purchased from Lonza, Cat. no. CC-2535 and cultured in EGM-2 medium according to manufacturer's protocol.

### Generation of iPSC Lines

Somatic reprogramming was used to generate iPSC lines from two healthy volunteers using the CytoTune-iPS 2.0 Sendai Reprogramming Kit according to the manufacturer's instructions. Two iPSC lines were generated from each volunteer and used for experiments. All procedures conformed to the UIC institutional review board-approved protocol.

### Differentiation of iPSC-ECs

Healthy iPSC-ECs (over passage 20) were split at a 1:12 ratio using EDTA and grown for 3–4 days until they reached ∼75% confluence. To initiate EC differentiation, the medium was changed to an induction medium (RPMI and B27-insulin, Thermo Fisher Scientific) supplemented with 6 μM CHIR on day 0 and 2 μM CHIR on day 2. For days 4–12, cells were cultured in different combinations of differentiation medium and EGM-2 medium from Lonza (100% differentiation medium on day 4, 50% differentiation medium and 50% EGM-2 on day 6, 25% differentiation medium and 75% EGM-2 medium on day 8, and 100% EGM-2 medium on day 10) with VEGF, FGF2, and BMP4 (PeproTech). At day 12 post-differentiation, cells were sorted using the human CD144 (VE-Cadherin) MicroBeads and magnetic cell sorting system (cat. no. 130-097-857, Miltenyi Biotech), as directed by the manufacturer, and expanded on 0.2% gelatin-coated plates. iPSC-ECs were then cultured in the EGM-2 medium at 37°C and 5% CO_2_ in a humidified incubator with medium changes every other day. Experiments described in this manuscript were performed between passages 2 and 4.

For comparison purposes, iPSC-ECs were also generated using an alternative protocol as previously described and named iPSC-EC NCB (Patsch et al., 2015). In brief, iPSCs were plated at a low density with Y-27632. The next day, differentiation was initiated by the addition of 8 μM CHIR and 25 ng/mL BMP4, and the medium was left unchanged for 3 days. On days 4 and 5, cells were cultured in StemPro-34 medium supplemented with 200 ng/mL VEGF and 2 μM forskolin, and the medium was replaced daily. Cells were then magnetically sorted using the human CD144 MicroBeads on day 6.

### Exposure of ECs to Hyperglycemia

iPSC-ECs (passages 2 to 4) and HAECs were seeded on 0.2% gelatin-coated plates in EGM-2 medium for 24 h. For hyperglycemic ECs, plating medium was then exchanged into a modified EGM-2 medium containing 33 mM glucose and 10 nM endothelin-1, and cultured for 72 h. For normoglycemic ECs, regular EGM-2 medium was used with the addition of mannitol as osmotic control. MDL-28170 was used at a final concentration of 2.5 μM where indicated.

### RNA Sequencing Analysis

The sequenced reads were aligned to human genome hg38 by HISAT2 (https://ccb.jhu.edu/software/hisat2/index.shtml). We used featureCounts (http://bioinf.wehi.edu.au/featureCounts/) to count the raw reads with the genome annotation Ensembl v.85. We then used DESeq2 of Bioconductor (https://bioconductor.org/packages/release/bioc/html/DESeq2.html) to normalize the raw counts, and generate a table of differentially express genes (DEGs) (likelihood ratio test, p < 0.05). The general linear model of the test: exp∼〖Treat〗_Glu+〖Treat〗_MDL+〖Treat〗_Glu:〖Treat〗_MDL, and 〖Treat〗_Glu:〖Treat〗_MDL is the interaction term of the two factors. Hierarchical clustering was implemented on the DEGs. Function enrichment analyses of the clusters of the DEGs were implemented by GeneAnswers of Bioconductor (https://www.bioconductor.org/packages/release/bioc/html/GeneAnswers.html).

### Statistical Analysis

All values are expressed as means ± SEM. Data were analyzed by one-way ANOVA followed by a Tukey multiple-comparison *post hoc* test. Differences were considered significant at values of p < 0.05.

## Author Contributions

S.-B.O., W.H.L., K. Kodo, H.R.J., and S.-G.O. designed and implemented the study and drafted the manuscript. S.-B.O. performed the mitochondrial and IR-related studies. W.H.L. performed the characterization of the iPSC-ECs and the autophagy studies. S.-B.O., W.H.L., N.I.I., M.-M.L., K. Katwadi, X.-Y.K., N.A.M., J.L., J.N., S.T., J.R., and N.-Y.S. were involved in data acquisition and data analysis. All authors reviewed and approved the final manuscript.

## Accession Numbers

RNA sequencing data can be accessed at NCBI GEO datasets using the accession number GSE123699.

## Supplemental Information

Document S1. Supplemental Experimental Procedures and Figures S1–S3

Document S2. Article plus Supplemental Information
