# Supplementary material for: Calpain Inhibition Restores Autophagy and Prevents Mitochondrial Fragmentation in a Human iPSC Model of Diabetic Endotheliopathy
Source: Stem Cell Reports. 2019 Feb 21;12(3):597–610. doi: 10.1016/j.stemcr.2019.01.017 (PMC6411483; doi:10.1016/j.stemcr.2019.01.017)
Supplement: Document S1. Supplemental Experimental Procedures and Figures S1–S3 [file mmc1.pdf]

Stem Cell Reports, Volume *12*

Supplemental Information

Calpain Inhibition Restores Autophagy and Prevents Mitochondrial Fragmentation in a Human iPSC Model of Diabetic Endotheliopathy

Sang-Bing Ong, Won Hee Lee, Ning-Yi Shao, Nur Izzah Ismail, Khairunnisa Katwadi, Mim-Mim Lim, Xiu-Yi Kwek, Nathaly Anto Michel, Jiajun Li, Jordan Newson, Soroush Tahmasebi, Jalees Rehman, Kazuki Kodo, Hye Ryoun Jang, and Sang-Ging Ong

Supplemental Information

Calpain Inhibition Restores Autophagy and Prevents Mitochondrial Fragmentation in a Human iPSC Model of Diabetic Endotheliopathy

Sang-Bing Ong, Won Hee Lee, Ning-Yi Shao, Nur Izzah Ismail, Khairunnisa Katwadi, Mim-Mim Lim, Xiu-Yi Kwek, Nathaly Anto Michel, Jiajun Li, Jordan Newson, Soroush Tahmasebi, Jalees Rehman, Kazuki Kodo, Hye Ryoun Jang, Sang-Ging Ong

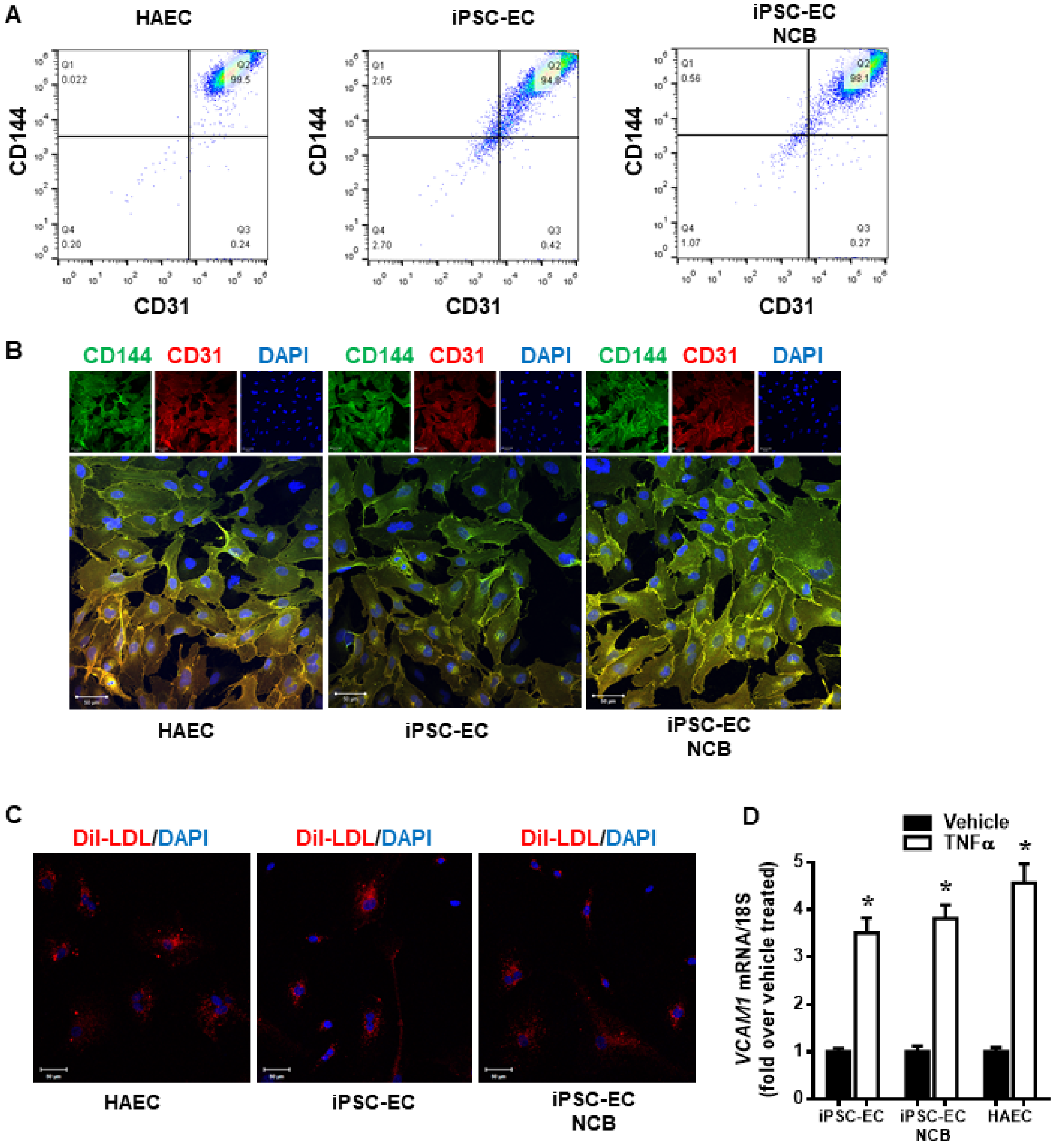

Figure S1. Characterization of iPSC-ECs from two differentiation protocols. Related to Figure 1. (**A**) Representative FACS analysis comparing purified iPSC-ECs from this manuscript vs. iPSC-ECs differentiated from an alternative protocol (iPSC-NCB, Patsch et al., 2015) vs. human aortic endothelial cells (HAEC) as a positive control. iPSC-ECs from both protocols which were isolated using CD144 MicroBeads were also positive for CD31, a commonly used EC marker. (**B**) iPSC-ECs from both differentiation protocols stained positive for both CD31 (red) and CD144 (green). Scale bar 50 µm. (**C**) Representative fluorescence images comparing uptake of acetylated LDL (red) in iPSC-ECs from both differentiation protocols with HAECs. Cells were counter-stained with DAPI (blue); scale bar 50 µm. (**D**) qPCR of vascular cell adhesion molecule 1 (*VCAM1*) following tumor necrosis factor alpha (TNFα) (10 ng/ml) treatment for 6 hours in iPSC-ECs compared to HAECs. Data are represented as mean ± SEM (N=4 independent experiments, *P<0.05 vs. vehicle-treated cells).

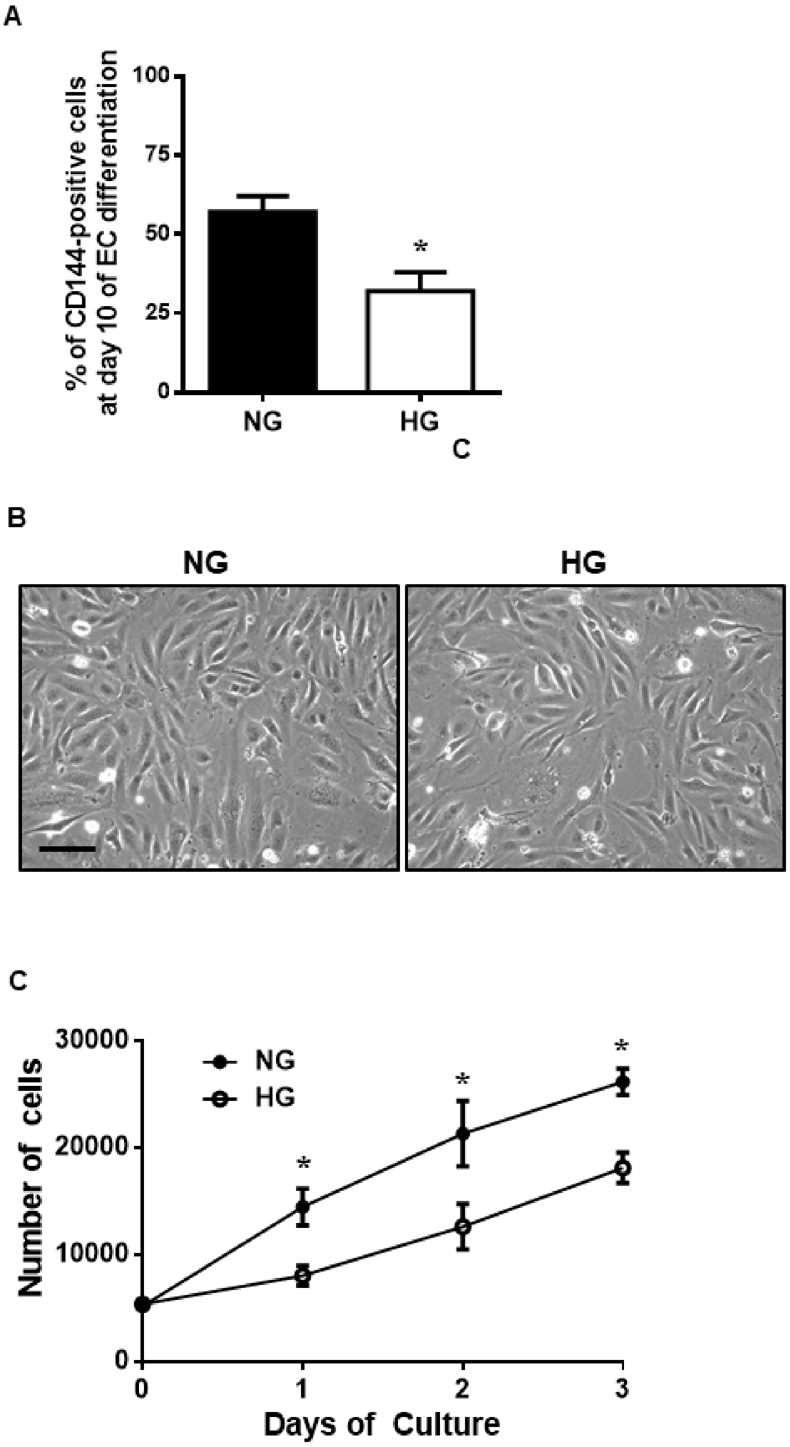

Figure S2. **Effects of hyperglycemia on differentiation and proliferation potential of iPSC-ECs. Related to Figure 2**. (**A**) Proportion of CD144-positive endothelial cells following 10 days of either normoglycemia (NG) or hyperglycemia (HG) treatment of iPSCs during differentiation. (N=4 independent experiments, *P<0.05 vs NG cells). (**B**) Representative bright-field images of differentiated iPSC-ECs cultured in either NG or HG conditions for 48 hours. Scale bar 100 µm. (**C**) Proliferative capacity of iPSC-ECs cultured over 3 days in either NG or HG conditions. Data are represented as mean + SEM (N=4 independent experiments, *P<0.05 vs NG cells).

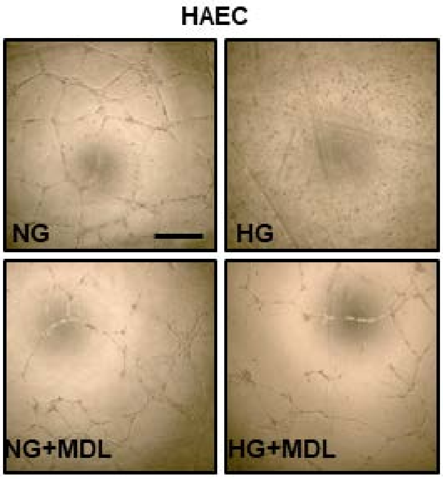

Figure S3. Representative images of HAECs exposed to normoglycemia or hyperglycemia with and without MDL-28170 treatment. Scale bar 100 µM. Related to Figure 3A.

Supplemental Experimental Procedures

Characterization of iPSC-ECs. Flow cytometry analysis, immunostaining, and qPCR were performed to assess endothelial differentiation efficiency and to confirm endothelial cell phenotypes. For flow cytometry, the cells were stained with FITC-conjugated CD31 antibody (Cat#:555445; BD Biosciences) and PE-conjugated CD144 antibody (Cat#: 560410) for 15 minutes in the dark at 4°C and analyzed using an LSRII flow cytometer (BD Biosciences). Isotype controls (PE-mouse IgG1,κ; Cat#: 555749 and FITC-mouse IgG1,κ; Cat#: 555748, BD Biosciences) were used to establish gating (data not shown). Data analysis was conducted using the FlowJo software (Three Star). For immunostaining, the cells were fixed in 4% paraformaldehyde (PFA) for 10 minutes, permeabilized in 0.1% triton-X, and blocked with 10% normal donkey serum in PBS for an hour. The cells were then incubated with the primary antibodies (CD31; Cat#: AF806 and CD144; Cat#: AF938 both from R&D Systems) in 5% normal donkey serum in PBS overnight at 4°C. After three washes with PBS, cells were incubated with secondary antibodies (Donkey anti-sheep; Cat#: A11015 and Donkey anti-goat; Cat#: A11058, Thermo Scientific) and DAPI. Images were acquired with a fluorescence microscope. For gene expression analysis, total RNA was isolated from iPSC-ECs with RNeasy Mini Kit (Qiagen) per manufacturer's instructions. RNA was reverse-transcribed into cDNA using High-Capacity cDNA Reverse Transcription Kit with RNase Inhibitor (ThermoFisher). Quantitative PCR was performed using TaqMan Universal PCR Mix (ThermoFisher) on a StepOnePlus Real-Time PCR system (ThermoFisher) with TaqMan primers against *CD31*, *CD144*, *vWF*, *VCAM-1*, and *GAPDH*. Expression of *GAPDH* was used as internal controls to normalize expression of the genes of interest. Relative gene expression is shown as a fold-change relative to the control group, averaged from three independent experiments. For assessing the uptake of acetylated low-density lipoprotein (LDL), iPSC-ECs were incubated with 10 μg/ml fluorescently-labeled LDL (ThermoFisher; Cat. # L3484) for 4 hours, fixed and imaged using fluorescence microscope. All the above experiments were also performed on HAECs in a similar method for comparison. For measurement of ECs proliferation, iPSC-ECs were seeded in 96-wells plates at a density of 5,000 cells per well under desired conditions. Proliferation was then quantified using the CyQUANT® Direct Cell Proliferation Assay according to manufacturer's protocol.

Tube formation assay. In vitro tube formation assay was performed per the manufacturer's instructions. Briefly, after coating the 15-well μ-Slide Angiogenesis (Ibidi GmbH), iPSC-ECs or HAECs were seeded at 10 000 cells/well. After 16 hours, capillary network images were taken using a Revolve microscope and quantitation was performed using ImageJ.

Assessment of autophagy levels. iPSC-ECs and HAECs lysates were prepared using RIPA buffer supplemented with protease-phosphatase inhibitor cocktail. Samples were incubated with either Beclin-1 antibody (Cat#: 4122S, Cell Signaling), LC3 antibody (Cat#: 4108S, Cell Signaling) or GAPDH (Cat#: 2118S, Cell Signaling), and secondary antibodies (anti-mouse or rabbit IgG; Cat #: 7076S and 7074S) conjugated with horse radish peroxidase (Cell Signaling). Densitometry analysis was performed using ImageJ software. Staining of autophagosomes were also performed in iPSC-ECs using the CYTO-ID Autophagy detection kit 2.0 (Enzo Life Sciences) according to manufacturer's instructions.

Cellular assays for ATP, ROS, caspase 3/7, and calpain activity. Following treatment of iPSC-ECs and HAECs under normoglycemic or hyperglycemic conditions with or without MDL-28170, 100 µl of CellTiter-Glo 2.0 (Promega) reagent was added directly to each well of 96-well plates, followed by incubation at room temperature on an orbital shaker to induce cell lysis for 12 minutes. For assessing intracellular ROS generation, six hours prior to the completion of treatment the H2O2 substrate was added and incubated for the final 6 hours of the experiment. 50 µl of media samples were transferred to a separate 96-well white-sided plate and 50 µl of ROSGlo™ Detection Solution was added to each well, followed by incubation at room temperature for 20 minutes before detecting luminescence signal. Cells in the original sample plate were kept for measuring total cell number by Calcein-AM (Invitrogen) to allow normalization. In addition, the activity of caspase 3/7 was assessed after adding 100 µl of Caspase-Glo 3/7 reagent to each well for an hour and total luminescence was measured. Calpain activity was measured using Calpain-Glo Protease Assay (Promega, G8501) according to manufacturer's instructions.

Assessment of mitochondrial morphology. Mitochondrial morphology was determined in iPSC-ECs and HAECs stained with MitoTracker Red (MTR) using a Leica TCS SP8 confocal microscope equipped with 63× oil immersion objective (Plan-Apochromat, NA 1.3) (Ong et al., 2010, 2014). Eighty randomly chosen cells per treatment group were designated as containing either predominantly (>50%) elongated or predominantly (>50%) fragmented mitochondria by 3 investigators blinded to the treatment. Quantitative analysis of mitochondrial morphology was performed on the confocal images using the Fiji/ImageJ software. The images were processed, binarized and subjected to particle analysis to generate characteristics of mitochondrial morphology: aspect ratio (AR, ratio between the major and minor axis of the ellipse equivalent to the mitochondrion) to estimate the length and circularity (ratio between their area and perimeter) to determine the degree of branching of the mitochondria (Juge et al., 2016).

iPSC-ECs cell death after sIRI. To determine the effect of calpain inhibition on the susceptibility to SIRI, iPSC-ECs were subjected to 6 hours of simulated ischemia in an air-tight hypoxic chamber followed by 24 hours of simulated reperfusion, at the end of which cell death was assessed by propidium iodide staining (Ong et al., 2010, 2014). For each treatment group, 80 cells taken from 4 randomly selected fields of view were counted. This experiment was repeated on at least 4 separate occasions.

References

Patsch, C., Challet-Meylan, L., Thoma, E.C., Urich, E., Heckel, T., O'Sullivan, J.F., Grainger, S.J., Kapp, F.G., Sun, L., Christensen, K., et al. (2015). Generation of vascular endothelial and smooth muscle cells from human pluripotent stem cells. Nat Cell Biol 17, 994-1003.

Juge, R., Breugnot, J., Da Silva, C., Bordes, S., Closs, B., and Aouacheria, A. (2016). Quantification and Characterization of UVB-Induced Mitochondrial Fragmentation in Normal Primary Human Keratinocytes. Sci Rep *6*, 35065.
